# Quantification of Fat Metaplasia in the Sacroiliac Joints of Patients With Axial Spondyloarthritis by Chemical Shift-Encoded MRI: A Diagnostic Trial

**DOI:** 10.3389/fimmu.2021.811672

**Published:** 2022-01-18

**Authors:** Dong Liu, Churong Lin, Budian Liu, Jun Qi, Huiquan Wen, Liudan Tu, Qiujing Wei, Qingcong Kong, Ya Xie, Jieruo Gu

**Affiliations:** ^1^ Department of Rheumatology, The Third Affiliated Hospital of Sun Yat-Sen University, Guangzhou, China; ^2^ Radiology Department, The Third Affiliated Hospital of Sun Yat-Sen University, Guangzhou, China

**Keywords:** axial spondyloarthritis, magnetic resonance imaging, fat metaplasia, chemical shift-encoded sequence, quantitative imaging

## Abstract

**Objective:**

To study the diagnostic performance of chemical shift-encoded MRI (CSE-MRI) in the diagnosis of axial spondyloarthritis (axSpA).

**Methods:**

CSE-MRI images were acquired for consecutive patients complaining of back pain as well as healthy volunteers. Proton density fat fraction (PDFF) values were measured independently by two readers. Diagnostic performance of CSE-MRI was analyzed by sensitivity analysis and ROC curve analysis. Logistic regression analysis was employed to investigate the risk factors of extensive fat deposition in the SIJs.

**Results:**

A total of 52 r-axSpA patients, 37 nr-axSpA patients, 24 non-SpA patients and 34 healthy volunteers were included. Mean PDFF values in the SIJs of patients with r-axSpA and nr-axSpA (72.7% and 64.5%) were significantly higher than non-SpA patients and healthy volunteers (56.0% and 57.6%) (p<0.001). By defining extensive fat deposition in the SIJs as ≥8 ROIs with PDFF values over 70%, its sensitivity and specificity in diagnosing axSpA reached 72.47% and 86.21%%. By joining bone marrow edema (BME) with ≥8 ROIs (PDFF>70%), 22 (24.71%) and 23 (25.84%) more axSpA patients were classified as SIJ MRI (+) by reader 1 and 2, but specificities decreased by 15.52% and 10.34%. Multivariate logistic regression analysis confirmed longer disease duration as the independent risk factor of extensive fat deposition in SIJs (OR=1.15, 95%CI[1.03, 1.32]), while bDMARDs medication was a protective factor (OR=0.15, 95%CI[0.04, 0.51]).

**Conclusion:**

CSE-MRI is a reliable tool to quantitively assess the fat metaplasia in the SIJs of axSpA patients. Extensive fat deposition in the SIJs could add incremental diagnostic value to BME, but at the cost of decreased specificities.

## Introduction

Quantitative imaging emerges as one of the major breakthroughs in medical imaging over the last two decades. Various quantitative imaging sequences have been successfully incorporated in the daily practice of disease diagnosis and therapeutic response monitoring, such as apparent diffusion coefficients (ADC) in hyperacute stroke (1, 2) and signal intensity ratio (SIR) in hepatic iron assessment (3, 4). Alas, the imaging diagnosis of axial spondyloarthritis (axSpA) still heavily depends on traditional MRI sequences, such as short tau inversion recovery (STIR) or T2-weighted fat-suppressed imaging (T2-FS), and T1-weighted imaging (T1WI) (5). Despite the fact that a variety of MRI lesions could be observed in the sacroiliac joints (SIJs) of patients with axSpA, the current definition of a positive SIJ MRI still remains indispensably the bone marrow edema (BME) or osteitis (6, 7). Still, a subset of patients highly suspected of axSpA do not exhibit such distinct inflammation in the SIJs on MRI, and could only be classified as axSpA through the clinical arm (8).

Fat metaplasia has been gaining interests in recent years regarding its capability of assisting in the imaging diagnosis of axSpA as well as predicting radiographic progression. Bakker et al. suggested that fat depositions and erosions combined could be used reliably as a substitute for radiographs in the imaging arm of ASAS classification criteria for axial spondyloarthritis (9). In a most recent study, fat lesions showed the highest specificity in the diagnosis of axSpA, compared with other lesions such as bone marrow edema and erosions, despite relatively low sensitivity (10). Whilst fat metaplasia displayed outstanding capability in the diagnosis of axSpA, it had also been suggested that fat metaplasia could be the intermediate link between inflammation and new bone formation (11, 12). A couple of studies revealed that fat metaplasia developed ensuing the resolution of inflammation, and such fatty lesions were independently associated with the development of ankylosis (13, 14).

Iterative Decomposition of water and fat with Echo Asymmetry and Least squares estimation (IDEAL-IQ), a chemical shift-encoded (CSE) sequence, is considered the state-of-the-art technique in fat quantification (15, 16). Compared with other sequences such as T1-in-and-out-of-phase (IOP) MRI, CSE-MRI is a more sophisticated fat quantification approach which corrects for a number of confounding factors including T1 bias, noise bias and eddy currents (17–19). An iterative least-squares decomposition algorithm is used to solve for proton density fat fraction (PDFF) maps enabling the quantitative assessment of fatty lesions, which have been widely applied in the imaging of non-alcoholic fatty liver disease (20, 21) as well as bone marrow fat in hematological diseases (22).

This study intended to conduct a diagnostic trial of CSE-MRI in the SIJs of patients with axSpA. It was hypothesized that the fat fraction in the SIJs in axSpA patients was significantly higher than non-spa patients and healthy controls. Additionally, it was also hypothesized that CSE-MRI could provide incremental diagnostic power to BME alone.

## Methods

### Study Population and Diagnosis

Consecutive patients complaining of back pain were included in this study when presenting to the rheumatology clinic at the Third Affiliated Hospital of Sun Yat-sen University between December 1st, 2020 and October 4th, 2021. Exclusion criteria included patients older than 50 years old, BMI>30kg/m^2^, accompanied by metabolic syndromes or malignant tumor. All study subjects received a complete diagnostic workup, with laboratory tests of C reactive protein and HLA-B27 as well as imaging assessments including MRI and radiographs. A board of two rheumatologists with clinical experience over 10 years (J.Q. and L.T) and a radiologist with clinical experience over 8 years (Q.K.) agreed upon the diagnosis of each patient as axSpA or non-SpA based on clinical data and imaging exercises. After receiving the diagnosis, patients were further classified as r-axSpA or nr-axSpA according to the 2009 ASAS classification criteria for axSpA (7).

Healthy volunteers with no previous history of back pain, other rheumatological conditions, malignant tumor or obesity (BMI>30kg/m^2^) were recruited to be examined with the same MRI protocol. Age and BMI of healthy volunteers were comparable to the included patients with back pain.

Clinical parameters such as age, sex, body mass index (BMI), disease duration, smoking history, BASDAI, ASDAS-CRP and bDMARDs medication history were recorded in detail.

### Imaging Procedures

All patients underwent MRI scanning of the sacroiliac joints in the supine position using a 3.0 T superconducting MR scanner (SignaTM Architect, GE Healthcare, Milwaukee, WI) with an anterior 30-channel and posterior 40-channel adaptive image receive (AIR) radiofrequency coil. The routine SIJ MRI examination consisted of T2-weighted fat-suppressed turbo spin echo sequence (T2-FS), T1-weighted images sequence (T1WI), T1-weighted images with fat saturation (T1-FS) sequence in a semi-coronal orientation and T2-FS sequences in a semi-axial orientation for the SIJ were available. The chemical shift-encoded sequence in the semi-coronal orientation of the SIJ were acquired under the following scan parameters: TR=12.1 ms. TE=6 ms; bandwidth = 142.86 KHZ; echo train length (ETL) = 3; flip angle=5;number of excitations (NEX)=1; matrix 256x256; 24 sections at a thickness of 4 mm; and scan time= 2 minutes 40 seconds.

### Fat Fraction Estimation

Two observers independently evaluated the CSE-MRI images, blinded to the patient information and diagnosis. The two observers included one junior rheumatologist (D.L.) and one senior radiologist (C.L.). The CSE-MRI images were all anonymized and presented to the observers in a random order.

**Figure 1:**
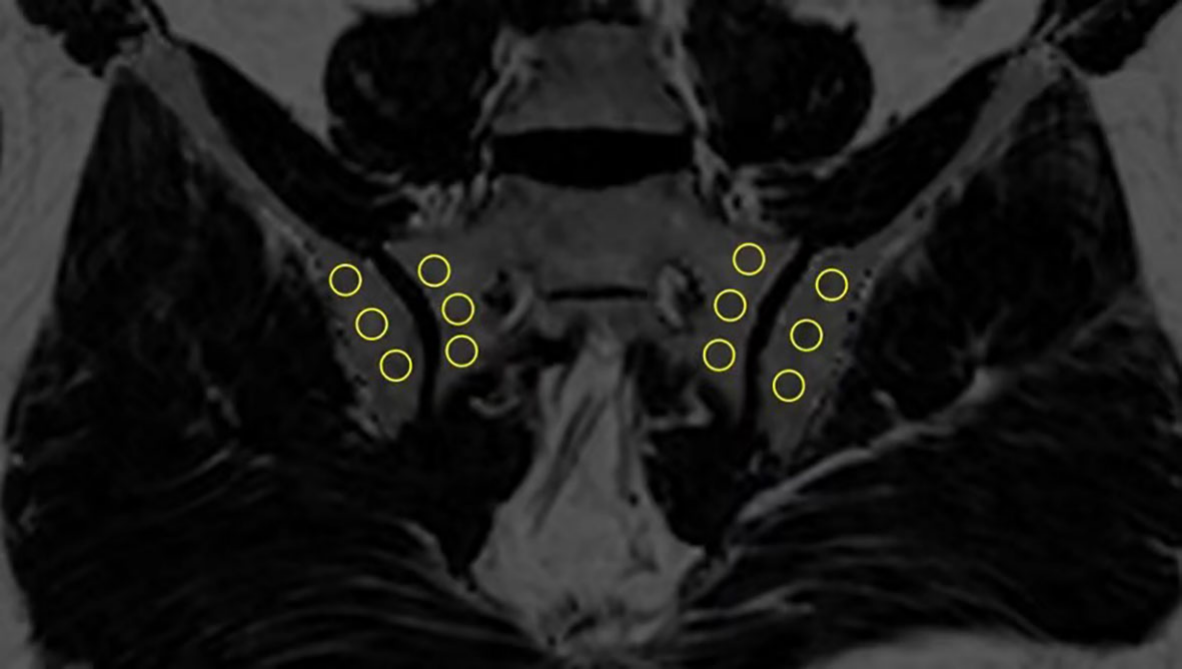
Placement of ROIs in the subchondral area in the SIJs (ROI, regions of interest; SIJs, sacroiliac joints).

In order to estimate the fat fraction of each participant in the SIJs, the fat fraction maps were presented to each observer. Three consecutive slices most representative of the sacroiliac joint in the semi-coronal orientation were selected for the delineation of regions of interest (ROI). ROIs come in the shape of circles with sizes ranging from 10-40 mm^2^. Three ROIs were manually placed in the subchondral bone on both the left and right sacral side and iliac side (**Figure 1**). The overarching principle for ROI placement is that ROIs should be evenly distributed while capturing the extent of regional fat deposition. ROIs should stay clear of any erosion, blood vessel, cavity or obvious regional bone marrow edema. The mean PDFF values within the ROIs were generated automatically by the system.

### Statistical Analysis

All statistical analysis was performed in software packages R version 3.6.3. Characteristics of study subjects were summarized using descriptive statistics. Repeated-measurements analysis of variance was employed to analyze the PDFF differences in each subcategories. Intergroup analysis was conducted according to different diagnostic groups, sex, age groups, HLA-B27, disease activity status, disease duration, bDMARDs medication history, smoking history, BMI. Furthermore, a pair-wise comparison was conducted to analyze the differences of PDFF values in different diagnostic groups.

Sensitivities, specificities, positive predictive values (PPV) and negative predictive values (NPV) were calculated for each cut-off point of mean PDFF values and counts of ROIs with PDFF values over 70%. The Youden index was employed to select the most appropriate cut-off point. Area under the curve (AUC) were also calculated.

The following candidate definitions of a positive SIJ MRI were considered: 1)overall mean PDFF values; 2)counts of ROIs with PDFF values over 70%; 3)BME combined with overall mean PDFF values; 4)BME combined with counts of ROIs with PDFF values over 70%; 5)BME alone. Receiver operating characteristic (ROC) curves were used to calculate the different levels of sensitivity and specificity at every cut-off point for 1) and 2). A comparison between all the candidate definitions was done to assess the incremental diagnostic value of CSE-MRI. Sensitivity analysis was conducted in all the included patients as well as in patients without previous bDMARDs medication. The intraclass correlation coefficient (ICC) was calculated to assess the 2 observers’ consistency.

By defining extensive fat deposition in the SIJ as ≥8 ROIs with mean PDFF values over 70% [≥8 ROIs(PDFF>70%)], logistic regression analysis was applied to explore the associations between extensive fat deposition and clinical parameters. Logistic regression analysis was also employed to examine the association between New York criteria (+) (mNY+) and extensive fat deposition in the SIJ.

## Results

### Descriptive Analysis

A total of 113 patients with back pain and 34 healthy controls were included in this study. Among the patients with back pain, 89 were diagnosed as axSpA and 24 were diagnosed as non-SpA patients. Based on the radiographs, 52 of the axSpA patients were further classified as r-axSpA while 37 were classified as nr-axSpA. Among patients with bDMARDs medication, 4 patients were treated with secukinumab, while 19 patients were treated with TNF-α inhibitors. Characteristics of participants were listed in **Table 1**.

**Table 1:** Characteristics of included participants.

Characteristics	Healthy volunteers (n=34)	Non-SpA (n=24)	nr-axSpA (n=37)	r-axSpA (n=52)	P-value
Age (years), mean ± SD	32.6 ± 8.36	30.3 ± 8.48	28.7 ± 7.49	31.6 ± 8.35	0.189
Male patients, n (%)	22 (64.71%)	8 (33.33%)	23 (62.16%)	45 (86.54%)	<0.001
BMI, kg/m2, mean ± SD	22.5 ± 3.22	21.3 ± 3.20	21.6 ± 2.72	22.3 ± 3.35	0.329
Disease duration (years), median [interquartile range]		1.5 [0.8-3]	1 [0.5-4]	6 [3-10]	<0.001
HLA-B27, n (%)		2 (8.33%)	31 (83.78%)	46 (88.46%)	<0.001
Smoking History, n (%)			4 (10.81%)	21 (40.38%)	<0.001
bDMARDs medication, n (%)			8 (21.62%)	15 (28.85%)	0.602
BASDAI (0-10), median [interquartile range]			1.70 [0.80-2.70]	3.15 [1.17-5.90]	0.002
ASDAS-CRP, median [interquartile range]			1.30 [0.98-2.31]	2.58 [1.80-3.29]	<0.001
SPARCC (0-72), median [interquartile range]			13.4 [0-26]	17.9 1.75-31.2]	<0.001
SSS, median [interquartile range]			27.4 [16.5-35.5]	56.5 [46.8-72.2]	<0.001

SpA, spondyloarthritis; nr-axSpA, non-radiographic spondyloarthritis; r-axSpA, radiographic spondyloarthritis; bDMARDs, biologic disease-modifying antirheumatic drugs; BASDAI, Bath Ankylosing Spondylitis Disease Activity Index; ASDAS-CRP, Ankylosing Spondylitis Disease Activity Score with C-reactive protein; SPARCC, Spondyloarthritis Research Consortium of Canada; SSS, MRI Sacroiliac Joint Structural Score.

### Quantitative Analysis of Fat Fraction

**Figure 2:**
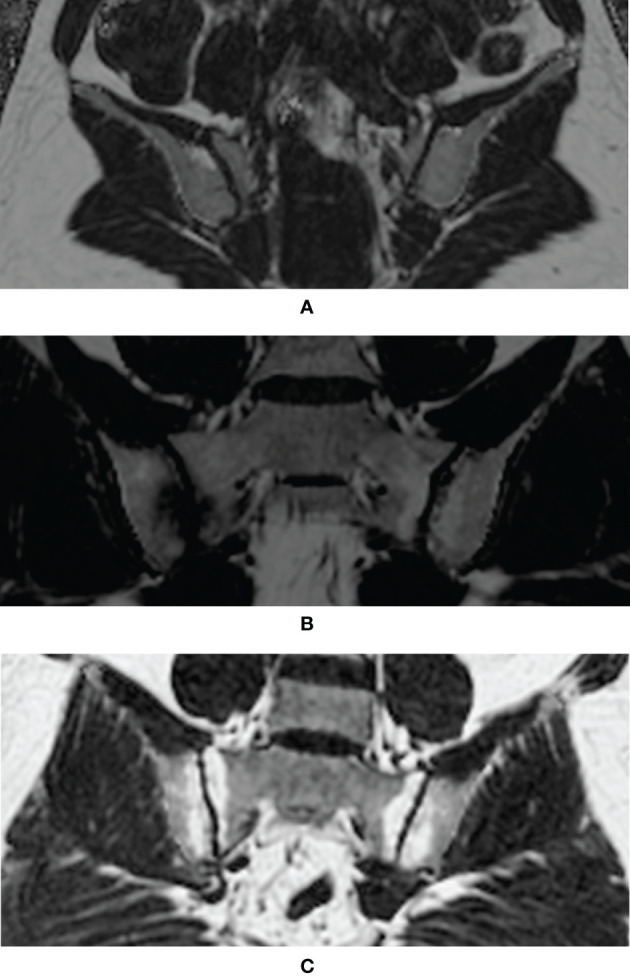
Examples of typical fat deposition patterns in different diagnostic groups. **(A)** Healthy volunteers with regional hot spots of fat deposition in the SIJ **(B)** SpA patients with simultaneous fat deposition and bone marrow edema in the SIJ **(C)** axSpA patients with fat deposition diffusely distributed in the subchondral area in the SIJ (SpA, spondyloarthritis; axSpA, axial spondyloarthritis; SIJ, sacroiliac joint).

**Figure 2** each displayed a typical example of the fat deposition pattern of: (A) Healthy volunteers with regional hot spots of fat deposition in the SIJ (B) SpA patients with simultaneous fat deposition and bone marrow edema in the SIJ (C) axSpA patients with fat deposition diffusely distributed in the subchondral area in the SIJ. Results of the comparative analysis of the differences of PDFF values among different groups were shown in **Table 2**.

**Table 2:** Intergroup analysis of differences of PDFF values in the SIJs.

Parameter and subcategory		N (%) of participants	PDFF values, mean ± SD	P-value
Diagnosis (n=147)	Healthy volunteers	34 (23.1%)	56.0 ± 10.5	<0.001
	Non-SpA	24 (16.3%)	57.6 ± 11.6	
	nr-axSpA	37 (25.2%)	64.5 ± 13.3	
	r-axSpA	52 (35.4%)	72.7 ± 15.6	
Age (n=147)	<25	34 (23.1%)	61.0 ± 15.5	0.030
	25-35	46 (31.3%)	63.8 ± 14.0	
	>35	67 (45.6%)	67.7 ± 15.6	
Sex (n=147)	Female	49 (33.3%)	58.4 ± 13.2	<0.001
	Male	98 (66.7%)	67.4 ± 15.1	
Disease duration (n=89)	<5	49 (55.1%)	64.8 ± 14.3	<0.001
	5-10	22 (24.7%)	76.4 ± 14.1	
	>10	18 (20.2%)	72.8 ± 14.7	
BMI (n=89)	<20	25 (28.1%)	65.6 ± 16.2	0.056
	20-24	43 (48.3%)	69.4 ± 14.2	
	>24	21 (23.6%)	73.5 ± 14.9	
HLA-B27 (n=89)	Negative	12 (13.5%)	67.0 ± 14.9	0.444
	Positive	77 (86.5%)	69.7 ± 15.2	
Smoking history (n=89)	No	64 (71.9%)	67.7 ± 15.3	0.026
	Yes	25 (28.1%)	73.5 ± 14.1	
bDMARDs medication (n=89)	No	66 (74.2%)	70.7 ± 14.8	0.057
	Yes	23 (25.8%)	65.3 ± 15.7	
ASDAS (n=89)	<1.4	26 (29.2%)	66.1 ± 14.5	0.208
	1.4-2.1	17 (19.1%)	69.8 ± 14.6	
	>2.1	46 (51.7%)	70.9 ± 15.6	

PDFF, proton density fat fraction; SpA, spondyloarthritis; nr-axSpA, non-radiographic spondyloarthritis; r-axSpA, radiographic spondyloarthritis; bDMARDs, biologic disease-modifying antirheumatic drugs; ASDAS, Ankylosing Spondylitis Disease Activity Score.

**Figure 3:**
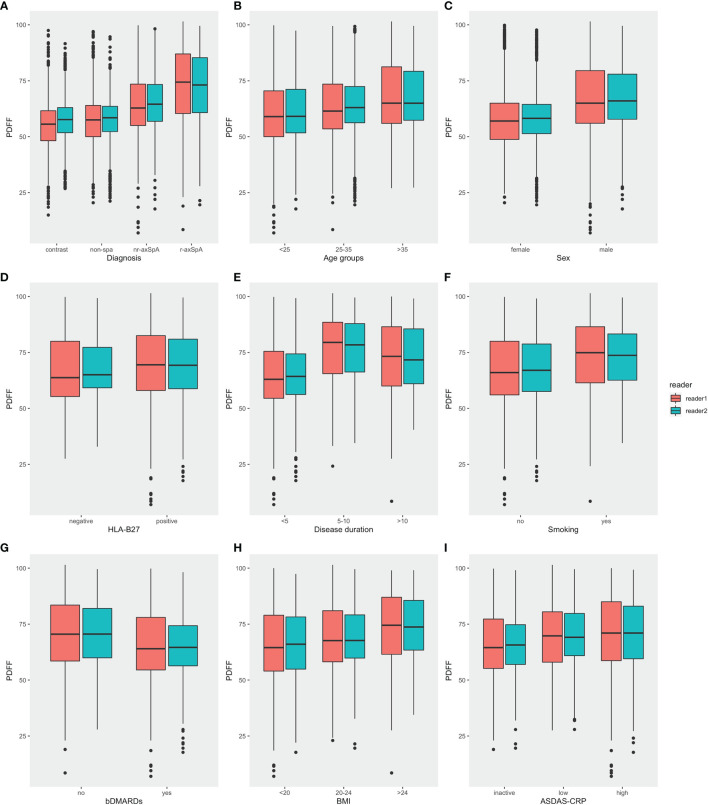
Comparisons of PDFF values in the SIJs in different categories. **(A)** Diagnosis **(B)** Age **(C)** Sex **(D)** HLA-B27 **(E)** Disease duration **(F)** Smoking history **(G)** bDMARDs medication **(H)** BMI **(I)** ASDAS (PDFF, proton density fat fraction; bDMARDs, biologic disease-modifying antirheumatic drugs; ASDAS, Ankylosing Spondylitis Disease Activity Score).

The foremost comparison was the analysis of PDFF values among different diagnostic groups, which revealed that there was a significant difference in the fat fraction among patients with different diagnoses (p<0.001). The mean PDFF values in the sacroiliac joints of the r-axSpA group were 72.7 ± 15.6%, while the mean PDFF values for nr-axSpA, non-SpA and healthy volunteers were 64.5 ± 13.3%, 57.6 ± 11.6% and 56.0 ± 10.5%, respectively. *Post-hoc* analysis of the different diagnostic groups further exhibited that the PDFF values of the r-axSpA group were significantly higher than the rest of the groups. The mean PDFF values in the nr-axSpA group, albeit lower than the r-axSpA group, were still significantly higher than non-SpA patients and healthy volunteers (**Table 3** and **Figure 3A**).

**Table 3:** Pair-wise analysis of differences of PDFF values in different diagnostic groups.

Comparison	Difference	P value
non-SpA - HC	1.59	0.9182
nr-axSpA - HC	8.55	0.0010
r-axSpA - HC	16.73	<0.0001
nr-axSpA - non-SpA	6.96	0.0238
r-axSpA - non-SpA	15.14	<0.0001
r-axSpA - nr-axSpA	8.18	0.0004

HC, healthy controls; SpA, spondyloarthritis; nr-axSpA, non-radiographic spondyloarthritis; r-axSpA, radiographic spondyloarthritis.

Moreover, male participants also presented significantly higher mean PDFF values compared with female participants. Mean PDFF values were also significantly higher in axSpA patients with an age >35 years old or with a disease duration > 10 years (**Table 2** and **Figure 3**).

### Diagnostic Performance of IDEAL-IQ

**Figure 4:**
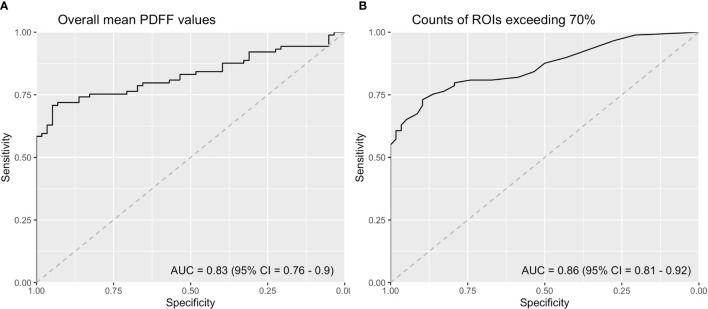
ROC curve analysis of **(A)** Overall mean PDFF values in the SIJs **(B)** Counts of ROIs with PDFF values exceeding 70% (ROI, region of interest; PDFF, proton density fat fraction).

ROC curve analysis demonstrated that the overall mean PDFF values had an AUC of 0.83 (95% CI = 0.76 - 0.90). Counts of ROIs with PDFF values over 70% reached an AUC of 0.86 (95% CI = 0.81 - 0.92) (**Figure 4**). Sensitivities, specificities and Youden indices of different overall mean PDFF values and counts of ROI (PDFF>70%) were listed in **Table 4**.

**Table 4:** Determination of cut-off levels for overall mean PDFF values and counts of ROIs (PDFF>70%).

Cut-off point	Overall mean PDFF values			Cut-off point	Counts of ROIs (PDFF>70%)		
	sensitivity	specificity	Youden index		sensitivity	specificity	Youden index
55.16	88.76%	31.58%	0.20	≥1	91.01%	31.90%	0.23
56.03	87.64%	38.60%	0.26	≥2	84.83%	50.00%	0.35
57.10	84.27%	43.86%	0.28	≥3	81.46%	61.21%	0.43
58.02	82.02%	54.39%	0.36	≥4	78.65%	65.52%	0.44
59.01	79.78%	59.65%	0.39	≥5	76.97%	68.97%	0.46
60.06	77.53%	68.42%	0.46	≥6	74.72%	75.86%	0.51
61.06	75.28%	71.93%	0.47	≥7	73.03%	80.17%	0.53
62.01	75.28%	82.46%	0.58	≥8	72.47%	86.21%	0.59
63.16	73.03%	87.72%	0.61	≥9	68.54%	87.07%	0.56
64.57	71.91%	91.23%	0.63	≥10	65.73%	88.79%	0.55
65.12	70.79%	96.49%	0.67	≥11	64.61%	90.52%	0.55
65.59	66.29%	96.49%	0.63	≥12	64.04%	92.24%	0.56
66.33	64.04%	96.49%	0.61	≥13	61.24%	93.97%	0.55
67.10	61.80%	96.49%	0.58	≥14	58.43%	97.41%	0.56
68.25	55.06%	100.00%	0.55	≥15	57.87%	98.28%	0.56
69.22	49.44%	100.00%	0.49	≥16	56.18%	98.28%	0.54
70.46	46.07%	100.00%	0.46	≥17	52.25%	99.14%	0.51
71.12	41.57%	100.00%	0.42	≥18	47.75%	99.14%	0.47
72.34	38.20%	100.00%	0.38	≥19	45.51%	99.14%	0.45
73.12	35.96%	100.00%	0.36	≥20	43.82%	99.14%	0.43

PDFF, proton density fat fraction; ROI, regions of interest.

BME alone only recognized a positive SIJ MRI in 59 (66.29%) and 56 (62.92%) axSpA patients for reader 1 and reader 2 respectively. By joining BME with ≥8 ROIs (PDFF>70%), 22 (24.71%) and 23 (25.84%) more axSpA patients were determined as SIJ MRI (+). However, specificities decreased by 15.52% and 10.34%. Sensitivities, specificities, PPV and NPV of all the candidate definitions in all the study subjects were listed in **Table 5**, while results of sensitivity analysis excluding patients with bDMARDs medication were listed in **Table 6**.

**Table 5:** Sensitivities and specificities of the 5 candidate definitions of a positive SIJ MRI in all the study subjects.

Reader	Definition	Healthy volunteers (n=34)	Non-SpA (n=24)	nr-axSpA (n=37)	r-axSpA (n=52)	Sensitivity	Specificity	PPV	NPV
Reader1	Mean PDFF values over 65%	2	3	19	39	65.17%	91.38%	92.06%	63.10%
	≥8 ROIs (PDFF>70%)	2	7	22	43	73.03%	84.48%	87.84%	67.12%
	BME or mean PDFF values over 65%	3	6	28	51	88.76%	84.48%	89.77%	83.05%
	BME or ≥8 ROIs (PDFF>70%)	4	10	30	51	91.01%	75.86%	85.26%	84.62%
	BME	2	3	18	41	66.29%	91.38%	92.19%	63.86%
Reader2	Mean PDFF values over 65%	2	4	19	38	64.04%	89.66%	90.48%	61.90%
	≥8 ROIs (PDFF>70%)	2	5	21	43	71.91%	87.93%	90.14%	67.11%
	BME or mean PDFF values over 65%	3	4	28	50	87.64%	87.93%	91.76%	82.26%
	BME or ≥8 ROIs (PDFF>70%)	3	5	28	51	88.76%	86.21%	90.80%	83.33%
	BME	1	1	18	38	62.92%	96.56%	96.55%	62.92%

SpA, spondyloarthritis; nr-axSpA, non-radiographic spondyloarthritis; r-axSpA, radiographic spondyloarthritis; BME, bone marrow edema; PDFF, proton density fat fraction; ROI, regions of interest; PPV, positive predictive values; NPV, positive predictive values.

**Table 6:** Sensitivities and specificities of the 5 candidate definitions of a positive SIJ MRI in study subjects excluding patients with bDMARDs medication.

Reader	Definition	Healthy volunteers (n=34)	Non-SpA (n=24)	nr-axSpA (n=29)	r-axSpA (n=37)	Sensitivity	Specificity	PPV	NPV
Reader1	Mean PDFF values over 65%	2	3	16	31	71.21%	91.38%	90.38%	55.79%
	≥8 ROIs (PDFF>70%)	2	7	19	33	78.79%	84.48%	85.25%	56.98%
	BME or mean PDFF values over 65%	3	6	22	37	89.39%	84.48%	86.76%	62.03%
	BME or ≥8 ROIs (PDFF>70%)	4	10	24	37	92.42%	75.86%	81.33%	61.11%
	BME	2	3	13	28	62.12%	91.38%	89.13%	52.48%
Reader2	Mean PDFF values over 65%	2	4	16	31	71.21%	89.66%	88.68%	55.32%
	≥8 ROIs (PDFF>70%)	2	5	17	33	75.76%	87.93%	87.72%	56.67%
	BME or mean PDFF values over 65%	3	4	22	36	87.88%	87.93%	89.23%	62.20%
	BME or ≥8 ROIs (PDFF>70%)	3	5	22	37	89.39%	86.21%	88.06%	62.50%
	BME	1	1	14	26	60.61%	96.55%	95.24%	53.33%

SpA, spondyloarthritis; nr-axSpA, non-radiographic spondyloarthritis; r-axSpA, radiographic spondyloarthritis; BME, bone marrow edema; PDFF, proton density fat fraction; ROI, regions of interest; PPV, positive predictive values; NPV, positive predictive values.

### The Inter-Observer Agreement

The inter-observer agreement was very good for the overall mean PDFF values [ICC=0.803, 95%CI (0.633, 0.884)] and excellent for ≥8 ROIs (PDFF>70%) [ICC=0.910, 95%CI (0.878, 0.935)].

### Association Between Extensive Fat Deposition in SIJs and Clinical Parameters

Univariate logistic regression analysis revealed significant associations between extensive fat deposition and age [OR=1.07, 95%CI (1.01, 1.13)], male patients [OR=3.23, 95%CI (1.18, 9.53)], disease duration [OR=1.16, 95%CI (1.06, 1.31)], bDMARDs medication [OR=0.25, 95%CI (0.09, 0.67)]. Multivariate logistic regression analysis confirmed longer disease duration [OR=1.15, 95%CI (1.03, 1.32)] as the independent risk factor of extensive fat deposition in the SIJs, while bDMARDs medication [OR=0.15, 95%CI (0.04, 0.51)] was the protective factor of extensive fat deposition (**Table 7**).

**Table 7:** Logistic regression analysis for the association between extensive fat deposition in the SIJs of axSpA patients and clinical predictors.

Parameter	Univariate analysis		Multivariate analysis	
	OR (95%CI)	P-value	OR (95%CI)	P-value
Age, years	1.07 (1.01,1.13)	0.027	1.04 (0.97, 1.12)	0.278
Sex (male *vs* female)	3.23 (1.18, 9.53)	0.026	2.15 (0.61, 7.99)	0.237
Disease duration, years	1.16 (1.06, 1.31)	0.005	1.15 (1.03, 1.32)	0.024
BMI, kg/m2	1.10 (0.96, 1.27)	0.175	–	–
Smoking history (yes *vs* no)	2.74 (1.04, 7.87)	0.049	1.37 (0.41, 4.75)	0.614
bDMARDs medication (yes *vs* no)	0.25 (0.09, 0.67)	0.008	0.15 (0.04, 0.51)	0.004
ASDAS	1.54 (1.05 2.37)	0.033	1.25 (0.79, 2.03)	0.349

### Association Between mNY(+) and Fat Fraction in SIJs

Univariate logistic regression analysis showed that there was a significant association between mNY(+) and extensive fat deposition in the SIJs (OR=1.08. 95% CI [1.02, 1.12]). However, multivariate logistic regression analysis failed to confirm extensive fat deposition in the SIJs as the independent risk factor of mNY(+) (OR=1.04, 95%CI [0.98, 1.10]). Instead, longer disease duration (OR=1.20. 95% CI [1.05, 1.41]) and higher ASDAS-CRP (OR=2.21, 95%CI [1.28, 4.11]) were identified as the independent risk factors of mNY(+) (**Table 8**).

**Table 8:** Logistic regression analysis for the association between fulfilling the modified New York criteria and clinical predictors.

Parameter	Univariate analysis		Multivariate analysis	
	OR (95%CI)	P-value	OR (95%CI)	P-value
Age, years	1.05 (0.99, 1.11)	0.093	–	–
Sex (male *vs* female)	3.91 (121, 10.33)	0.010	1.38 (0.38, 5.12)	0.627
Disease duration, years	1.30 (1.12, 1.54)	<0.001	1.20 (1.05, 1.41)	0.017
BMI, kg/m2	1.08 (0.96, 1.32)	0.265	–	–
Smoking history (yes *vs* no)	5.59 (1.38,22.53)	0.004	2.74 (0.70, 12.62)	0.164
bDMARDs medication (yes *vs* no)	1.47 (0.62, 4.85)	0.444	–	–
ASDAS	2.45 (1.44, 4.05)	<0.001	2.21 (1.28, 4.11)	0.007
Counts of ROIs (PDFF>70%)	1.08 (1.02, 1.12)	<0.001	1.04 (0.98, 1.10)	0.203

## Discussion

The CSE-MRI sequence is considered the state-of-the-art technique for the quantification of fat measurements (16). Its superiority to other fat quantification MR sequences, such as T1-in-and-out-of-phase (IOP) sequence, had been validated by a body of studies (17–19), even with evidence of histological analysis (23). Ren et al. led a preliminary effort to investigate CSE-MRI in the assessment of fat deposition in the SIJs of patients with ankylosing spondylitis (AS) (24). Yet, this study did not include patients without obvious structural damage on the radiographs, currently known as nr-axSpA, nor did it assess the incremental diagnostic values of fat fraction to BME, therefore alienating itself from daily clinical practice. A histographic study by Bray et al. established the connection between elevated PDFF values and the presence of fat metaplasia in the SIJs, thereby laying the foundation for our study (25). Our study was initiated from the daily clinical scenario, where patients complaining of back pain were classified as non-SpA, nr-axSpA and r-axSpA based on results of the diagnostic work-up. Results showed that the PDFF values in the SIJs of r-axSpA patients were significantly higher than the other three groups, which was in line with the previous study (24). On the other hand, the PDFF values in the SIJs of nr-axSpA patients, albeit lower than r-axSpA, were also significantly higher than the non-SpA group and healthy volunteers. An intriguing observation is that patients with axSpA exhibited different patterns of fat metaplasia in SIJs according to the fat fraction maps. For patients with long-standing sacroiliitis, fat deposition was often distributed in a diffuse fashion in the subchondral area in the SIJs (**Figure 2C**), leading to a high count of ROIs (PDFF>70%) (**Figure 2C**). Conversely, in some patients with acute sacroiliitis, due to the presence of intense bone marrow edema or osteitis, the PDFF values were lowered, while in other quadrants of the SIJs fat deposition was rather conspicuous (**Figure 2B**). This was also in line with the previous reports that fat deposition and acute bone marrow edema were mutually exclusive (26). Therefore, we held the opinion that the counts of ROIs with PDFF values over 70% might be a more reliable indicator than the overall mean PDFF values merely.

Noteworthy, regional hot spots of fat deposition could also be seen in non-SpA patients and healthy controls. A study by Baraliakos et al. observed that at least one fatty lesion in the vertebral corners was present in over 80% of the healthy volunteers (27). However, few healthy volunteers had more than 5 fatty lesions (27). Our study demonstrated that the regional hot spots of fat metaplasia notwithstanding, the fat fraction in SIJs was still relatively low in the non-SpA patients and healthy controls, with overall PDFF values approximately 50%.

Our study identified a longer disease duration as the independent risk factor of extensive fat deposition in the SIJs of patients with axSpA, while bDMARDs medication was identified as a protective factor. A previous study by Baraliakos et al. reported that fat deposition in the SIJs of a general population was significantly associated with an older age (27). Despite the fact that the mean PDFF values in the SIJs were significantly higher in patients with an older age as well as male patients, their association with fat deposition failed to be validated by the multivariate logistic regression analysis.

This study devised a viable scheme for the quantitative imaging tools to be incorporated in routine MR examinations for axSpA patients. Aiming at presenting the overall extent of fat metaplasia in the sacroiliac joints, this study adopted a sampling strategy instead of a whole-organ segmentation strategy. Common approaches to measuring quantitative imaging data include sampling strategy (28) and whole-organ segmentation strategy, with the latter one often applied in the assessment of organs in regular shapes, such as liver and pancreas (21, 29). In the case of the SIJs, the subchondral bone area come in an irregular shape, rendering the process of manually delineating the subchondral bone “painstaking”. ROIs in the shape of circles evenly distributed in the subchondral bone could manage to reflect the overall fat fraction while capturing the regional hot spots of fat deposition. The average time taken to measure the PDFF values for each patient ranges from 3-6 minutes, making this process much feasible in clinical practice. Another noteworthy strength of this fat quantification sequence lies in its robustness. Unlike semiquantitative scoring systems such as SSS, multiple training sessions were not essential for readers to measure the PDFF values. As indicated by its stellar inter-observer consistency, the assessment of PDFF values were reliable and easy to replicate.

CSE-MRI per se exhibited commendable diagnostic performance in the diagnosis of axSpA, while it could also provide incremental diagnostic value to BME. Approximately 10% more axSpA patients were identified if joining BME with CSE-MRI, indicating that fat quantification could be a reliable addition to the imaging diagnosis of axSpA. However, the elevated sensitivity comes with a price. Approximately 10-15% of healthy volunteers and non-SpA patients were classified as SIJ MRI (+) once we included ≥8 ROIs(PDFF>70%) in the definition. Deliberations were still warranted whether to include fatty lesions in the imaging arm of the classification criteria for axSpA, since fatty lesions were also common findings in healthy individuals. It should be noted that since the current imaging arm of the axSpA classification criteria rested on BME/osteitis to a great extent, some patients in a very early phase without obvious BME/osteitis could be classified as non-SpA. It was possible that some of these patients could evolve into full-blown SpA over time (30). Whether CSE-MRI could assist in the early diagnosis of this specific subset of patients required a longitudinal study to observe their disease progress.

Another ambition of this study was to examine the association between fat metaplasia and mNY(+). It has been established that fat metaplasia might be the key intermediaries between acute inflammation and new bone formation (11, 12). Machado et al. reported that MRI vertebral corner inflammation followed by fat deposition is the strongest contributor to the development of new bone at the same vertebral corner (31). Maksymowych et al. discovered that fat metaplasia in the SIJ could be the intermediate link in the development of SIJ ankylosis while also increasing the propensity for disease progression in the spine of patients with SpA (11, 12). In our study, multivariate logistic regression analysis failed to identify extensive fat deposition in the SIJs as the independent risk factor for mNY(+). However, caution must be taken to interpret this result. Since this study was a cross-sectional study, it would be far-fetched to assert whether fat metaplasia was a predictor of developing r-axSpA.

Whether TNF blockers could affect the radiographic progression in patients with axSpA remains a perennial discussion. The “TNF-brake hypothesis” has been a popular theory attempting to explain the dissociation between the improvement of disease activity and new bone formation (32, 33). Inflammation resolution achieved by TNF blockers could potentially give rise to the development of fatty lesions, which had been proved be to significantly associated with syndesmophytes. However, this theory also met with counter-arguments that continuous use of TNF blockers did not necessarily lead to an increased rate of new bone formation (13, 34, 35). Our study revealed that bDMARDs medication might be a protective factor for extensive fat deposition in SIJs. Yet again, no conclusions could be drawn given the cross-sectional nature of this study. More careful examination of the effect of bDMARDs on fat metaplasia in the axial skeleton was warranted.

The most important limitation to this study is its nature of a cross-sectional study. We did not prospectively follow the included patients, especially the ones currently classified as non-SpA, since some of the non-SpA patients could evolve into full-blown SpA over time. It was our intention, however, to conduct a longitudinal study to further assess the diagnostic value of CSE-MRI in the diagnosis of axSpA. Another limitation of our study was inclusion of patients previously medicated with bDMARDs. In our study, 23 patients were medicated with bDMARDs. Among them, 2 patients were treated for psoriasis and 3 patients were treated for inflammatory bowel diseases, while the rest had been treated for inflammatory back pain suspected of spondyloarthritis in other facilities, but due to poor responses to bDMARDs or other personal reasons requested another MRI examination seeking second opinions. Inclusion of patients with bDMARDs medication could supposedly underestimate the occurrence rates of BME, but by singling out patients without bDMARDs medication, we observed that the sensitivity of BME remained 60-70%. A most recent imaging study also reported that the sensitivity of BME was 72.5% (10). However, by including this part of patients, we were able to identify bDMARDs medication as a protective factor of extensive fat deposition in the SIJs.

In conclusion, CSE-MRI is a reliable tool to quantitively assess the fat metaplasia in the SIJs of patients with axSpA. Overall mean PDFF values in the SIJs of patients with r-axSpA and nr-axSpA were significantly higher than non-SpA patients and healthy volunteers. By defining extensive fat deposition in the SIJs as ≥8 ROIs with mean PDFF values over 70%, its sensitivity and specificity in diagnosing axSpA reached 72.47% and 86.21%. Quantitative assessment of fat deposition in the SIJs could provide incremental diagnostic value to BME, but at the cost of decreased specificities. Deliberation is still warranted whether to include fat deposition in the imaging arm of the ASAS classification criteria for axSpA.

## Data Availability Statement

The raw data supporting the conclusions of this article will be made available by the authors, without undue reservation.

## Ethics Statement

The studies involving human participants were reviewed and approved by the Ethical committee of the Third Affiliated Hospital of Sun Yat-sen University. The patients/participants provided their written informed consent to participate in this study.

## Author Contributions

DL and CL contributed equally to this manuscript. DL: idea, reading of images, and statistical analysis and drafting of the manuscript. CL: idea, reading of images, and devising reading protocol of the images. BL: blinding and management of images. JQ: determining diagnosis for patients. LT: determining diagnosis for patients. HW: image acquisition. QK: determining imaging diagnosis for patients. QW: collection of laboratory test results. YX: patient recruitment and study coordination. JG: idea and devising study protocol and editing of manuscript. All authors contributed to the article and approved the submitted version.

## Conflict of Interest

The authors declare that the research was conducted in the absence of any commercial or financial relationships that could be construed as a potential conflict of interest.

## Publisher’s Note

All claims expressed in this article are solely those of the authors and do not necessarily represent those of their affiliated organizations, or those of the publisher, the editors and the reviewers. Any product that may be evaluated in this article, or claim that may be made by its manufacturer, is not guaranteed or endorsed by the publisher.
